# Death from Rupture of the Semilunar Valves, with Commentary

**Published:** 1831-01-01

**Authors:** 


### Death from Rupture of one of the Semilunar Valves of the Aorta. By Dr. Plenderleath.

This case, being short, we shall copy it verbatim from our cotemporary, the Medical Gazette, for reasons to be adduced in the sequel.

"A. B. immediately after an examination before the College of Surgeons, most honourable to himself, complained of being generally unwell, with symptoms of indigestion; to dissipate which he was recommended to go to the coast, and was visited for the first time on the third of last month. At that time he had regained his appetite, but complained of a sense of fulness and of oppression about the chest, occuring in paroxysms, with despondency and symptoms almost amounting to complete hysteria. His pulse was full and strong, and he was recommended to lose blood. Having neglected this advice, in two days the symptoms became so aggravated that twenty ounces were taken from the arm with relief. The strength of the patient, however, becoming impaired, he was confined to bed with the above symptoms almost daily occurring, and more or less aggravated, especially those of hysteria. The pulse became intermitting, attended with a sudden jerk, which last symptom was particularly observable in the carotid arteries. His thirst was at no time much increased, and the urine only decreased in quantity within a week of the termination of life, and did not shew the presence of albumen. The stethoscope, used by an experienced practitioner, did not indicate the specific nature of any disease of the heart. The pulse, for three days before death, became regular, and on the morning of the second of October, when making an effort on the night-chair, the patient suddenly expired. During the three days preceding dissolution, the functions of the liver were suspended.

*Dissection.* Slight adhesions of the lining membrane of the chest; lungs much gorged with blood; pericardium contained four ounces of sero-sanguineous fluid, but shewed no signs of inflammation. The heart was of remarkable paleness; its substance very soft, much enlarged, and very thin, and so flabby as to make it difficult to examine it, the ventricles falling flat together. The right ventricle about one-third part of its usual thickness; the left ventricle very thin, and towards its apex remarkably so. The semilunar valves of aorta very much thickened, red, and in the tendinous margin loaded with calcareous matter. The appearance was as if studded with innumerable points, of the smallest possible size, like millet seeds; they were hard, and giving the feel of powdered bone. The appearance of the heart on first viewing it *in situ* was that of a diseased and thickened bladder; the substance tore very easily, suffering the fingers to be pushed through with great ease. The aorta reduced to half its size in diameter, and a good deal resembled parchment. In the right cavity of the chest a pint of sero-sanguineous fluid, including a large quantity of hydatids; in the left cavity a pint of similar fluid. One of the semilunar valves of the aorta was ruptured transversely to its base; the other valves healthy; stomach and liver healthy; spleen enlarged to twice its natural size; intestines healthy. In reviewing the above dissection, it is singular to find the left ventricle, which in the natural state is nearly twice the thickness of the right, to have lost so much more of its muscular structure, and to find that where the elasticity and contractility of the heart were so much diminished from the absorption of its cellular tissue and muscular fibre that rupture did not take place in its parietes, which were so much thinner and softer than in their state of healthy structure, but took place in a valve which had acquired a preternatural firmness."

With a highly diseased state of heart—an aorta contracted to half its natural calibre—a sero-sanguineous effusion into the pericardium—a disorganized condition of the aortic valves—a pint of sero-sanguineous fluid, including a quantity of hydatids, in each cavity of the chest—with all these lesions, we cannot but wonder why Dr. Plenderleath should pitch upon a rent in one of the semilunar valves as the cause of death? We hardly think that this rent had the slightest operative effect in the production of the final catastrophe. The office of the semilunar valves is to prevent the regurgitation of blood into the ventricle during the diastole. But the great difficulty, in this case, was to propel the blood through a contracted aorta by means of a softened and weakened heart.

---

